# Sculpting the microbiome

**DOI:** 10.1098/rstb.2023.0057

**Published:** 2024-05-06

**Authors:** Mark A. Hanson, Hannah E. Westlake, Catherine S. Schrankel

**Affiliations:** ^1^ Centre for Ecology and Conservation, University of Exeter, Penryn Campus, Penryn Cornwall TR10 9FE, UK; ^2^ Environment and Sustainability Institute, University of Exeter, Penryn Campus, Penryn Cornwall TR10 9FE, UK; ^3^ Department of Biology, San Diego State University, San Diego CA 92182, USA

**Keywords:** microbiota, microbiome, host–microbe, immunity, development, gut

## 1. Preface

Multicellular life evolved in the presence of microbes. These bacteria, fungi and viruses are an ever-present community known as the ‘microbiome.' Hosts have evolved to expect the presence of resident microflora, which are even required for the developmental programmes of some organisms. The microbiome further complements the metabolic needs of the host, who in turn promotes a particular assemblage of microbes. However, stress can change the character of these normally symbiotic relationships, leading to dysbiosis and negative health effects on the host. This presents a paradox for the host immune system: the host must expect and tolerate colonization by resident microbes, but must also remain vigilant against opportunistic infections by potential pathogens.

With the advent of CRISPR gene editing, single-cell RNA sequencing and low-cost genome sequencing, there has been a surge of valuable insights into how hosts select for and maintain microbial communities. Host–microbe interactions range across diverse animal phyla and microbial taxa. Traditionally, research in this area focused on how the presence/absence of the microbiome affects the host, or observed how immune or microbiome disruption affects the broad host–microbe system. In the last decade, the precision offered by modern techniques has helped to more finely decipher specific host mechanisms that sculpt and respond to the microbiome. There is a growing appreciation for how the microbiome provides a lens through which the host interprets its environment.^1^

This special issue reflects on recent discoveries that have changed the way we view host–microbiome interactions. Broadly, there are two major themes discussed throughout: first, modern techniques have now led to mechanistic understandings of host immune receptor and effector interactions with specific microbes. The power of the genetic tools and techniques of the last few years has provided confirmatory results for pre-existing ideas, but also a number of unexpected discoveries; and second, there has been increasing appreciation and precision in detailing the specialized niches that microbes colonize, and characterization of the metabolites microbes produce that impact the host. This interplay of host and microbe factors promotes healthy organism development and physiology through beneficial microbiome assemblages.

This special issue was inspired by the 2022 European Society for Evolutionary Developmental Biology conference session *‘*Animal immunity at the interface of development and the microbial environment'*.* It has benefitted from the input by authors from many research backgrounds studying host–microbe systems of diverse animal phyla. In total, the issue comprises 17 articles contributed by 65 authors from eight countries. Their professional expertise spans a range of study systems, including both classic animal model organisms and non-model species of interest. Their research covers diverse environments, including marine, freshwater and terrestrial organisms with an array of micro and macro niches from across the tree of life. As editors, we hoped to provide an article collection with a global view of host–microbe interactions, and we thank the authors for making that a reality.

## 2. Summary of articles

The issue begins with a broader reflection on host–microbe interactions. Animal hosts encounter an array of microbes in their environment that are stable across ecological and evolutionary timescales. As a consequence, evolutionary processes have established principles of host–microbe interactions that shape host immunity and development as well as microbiome composition and structure.

Hixson *et al*. [1] set the stage by detailing the diversity of host–microbe-immune interactions in mosquitoes. This group of insects is best known for transmitting parasites and viruses, and Hixson *et al*. [1] provide a holistic view of mosquito interactions incorporating bacterial and fungal associates to better understand the factors that shape mosquito development and immune activity. Hixson *et al*. [1] offer a unique clarity in understanding how host and microbe interact to develop the sum organism, which is all the more pertinent given the importance of mosquitoes to human health and disease.

Reflecting on host–virus interactions, Imler *et al*. [2] next synthesize recent work which has shown that even closely related species have distinct immune response profiles. Drawing a parallel from the hourglass model of evolutionary development (evo-devo), Imler *et al*. [2] describe ‘evo-immuno' dynamics. These dynamics act across multiple levels, from receptors to core signalling elements, which themselves regulate diverse effectors. Their article stresses that in order to understand the principles behind successful defence and microbial control, we must study not only what is conserved, but also what is unique.

Hanson [3] expands on this theme with a focus on host antimicrobial effectors. Recent work has found incredibly specific host effector–microbe interactions, first in *Drosophila* fruit flies and now in other model systems. Hanson [3] outlines the logical flow of immune signalling architecture, and uses this to explain effector activities in light of ‘the Achilles principle' of immune evolution. This ‘Achilles principle' is invoked to explain how hosts evolve the surprising effector specificity that has been repeatedly observed, but without triggering Red Queen host–microbe arms race dynamics.

These first papers draw heavily on insights from insect study systems, but there are plenty of things to FISH (fluorescence *in situ* hybridization; and other useful assays) in the sea. The following studies and reviews investigate organisms from marine and freshwater ecosystems. In these environments, hosts and symbionts share space in the sediment or water column. This environment yields a set of host–microbe interactions that are, quite literally, more fluid. However, hosts nevertheless find ways to adapt to and shape the microbiota around them.

Destomieux-Garzon *et al*. [4] reflect on the developmental and immune dynamics of the Pacific oyster. In the early stages of oyster life, the microbiome is key to the maturation of host immunity and intergenerational transfer of immune efficacy. This interplay of host immune effectors and the microbiome can be disrupted by disease, as emphasized by an ongoing viral pandemic that disrupts the balance of the microbiome and leads to dysbiosis and fatal sepsis. Maintaining this balance of host–microbe interactions is a challenging process requiring constant navigation, and recent technological advances have helped us appreciate the precise mechanisms underpinning these complex interactions.

The next article by Klimovich & Bosch [5] uses a mix of functional analysis, single-cell RNA sequencing and machine learning to detail the complexity of antimicrobial peptide contributions to microbiome maintenance in *Hydra* jellyfish. This unique model system allows detailed study of the gastric cavity in an organism with a functional and manipulable nervous system. Klimovich & Bosch [5] reveal how different endo- or ectodermal cell types produce varying complements of immune effectors, taking advantage of modern techniques. They discuss the actions of these antimicrobial peptides and Kazal-type protease inhibitors in the context of microbiome control.

On the theme of gastric cellular niches, Tran *et al*. [6] dissect the ability and importance of corals to select for algal symbionts using an extensive flow cytometry and confocal microscopy dataset. Coral growth depends on the partnership between the animal hosts and their intracellular, photosynthetic dinoflagellate (algal) symbionts. Tran *et al*. [6] investigate host pre- and post-phagocytic mechanisms that mediate discrimination, symbiotic establishment and maintenance of actively photosynthesizing algae in the sea anemone model *Aiptasia*. With this foundation, the nutritional impacts of photosynthetic dinoflagellate interactions can be extended to encompass algal–microbe interactions.

Working with sea urchins, Wessel *et al*. [7] next review how host-derived naphthoquinone pigments in the epidermis correlate with microbe colonization. Using CRISPR/Cas9 perturbation and 16S rRNA sequencing, Wessel *et al*. [7] highlight how localized differences in pigment production result in specific community structure of the surface microbiome. Molecular mechanisms of epithelial immunity, intra-lumen gut physiology or microbe–microbe interactions are often invoked in the establishment of the microbiome. This useful perspective reminds readers that seemingly passive aspects—such as organism colour—may shape or be shaped by roles beyond conventional ecological framing.

Stoeltje *et al*. [8] then take us inside emerging model systems to better understand host mechanisms that modulate entry and exit of small molecules (good or bad) generated by lumenal bacteria. ATP-binding cassette (ABC) transporters are present in all domains of life. Perturbation or loss of the exporter ABCB1/MDR1 in particular has been linked with heightened gut inflammation and dysbiosis. Decades of observations suggest a role in regulating the transit of bacterial compounds into or through gut epithelia. Stoeltje *et al*. [8] discuss how new genetic tools in zebrafish and sea urchins are well poised to study which bacteria-derived metabolites, toxins and/or other factors are substrates of host ABCB1. These new opportunities for study should provide insight to how ABC transporters modulate community structure, and in turn affect host inflammatory responses when perturbed.

Regulating the gut microenvironment is complex. In mammals, mucus binds and neutralizes pathogenic microbes, and host immunoglobulin (Ig)-based antibodies IgA and IgG also bind to mucins in the intestinal tract to facilitate retention and colonization of specific commensal microbes. Dishaw *et al.* [9] investigate similar interactions in the gut of marine invertebrate chordates (tunicates). Here, Ig-domain containing ‘V region-containing chitin-binding proteins' (VCBPs) tether to chitin-rich mucus of the gut, by way of their C terminal chitin-binding domain. Dishaw and colleagues illuminate how the binding of Ig domain effectors to the mucus matrix arose at least twice in chordate evolution. Thus, IgAs and VCBPs mediate convergent immune effector mechanisms that sculpt the composition of commensal communities colonizing the mucus lining epithelial surfaces.

These fundamental insights are made possible by the study of molecules common to diverse animals, even if their precise physiological context differs from organism to organism. Evolutionary developmental biology as a field has provided invaluable insights on principles common to all organisms.

Reflecting this view, Maritan *et al*. [10] provide a comprehensive review of how host factors shape and adapt to the presence of the microbiome across organisms. Their extensive discussion spans multiple animal phyla, existing in widely varying ecologies and reflects on the processes that are common or distinct to different lineages and different systems. They explore how host factors such as physiological gut niches and molecular mechanisms contribute to establishment and maintenance of the gut microbiome. This impressive effort is a valuable resource for any researcher interested in the fundamental principles of host–microbiome interactions.

Galambos *et al*. [11] similarly explores host-specific immune processes in symbiosis. The cereal weevil reflects the incredible extent to which hosts adapt to control and live in harmony with microbial symbionts. In cereal weevils, gut symbionts essential for healthy development are confined to specialized cells known as bacteriocytes. Detailed study and recent dual RNAseq of host and microbe shows that bacteriocytes both produce antimicrobial peptides (AMPs) to constrain the symbiont, but also protect the symbiont from circulating AMPs during an immune response. This model system details incredibly elegant mechanisms maintaining an obligate symbiosis, and how this has shaped host development, physiology and immunity.

One of the fundamental regions of host–microbe interactions is the gut. Ludington [12] provides a thoughtful exploration of how the gut niche space of diverse animal systems is the product of coevolution of both host and microbe. These host–microbe relationships have developed to both provide the host with advantageous nutrients, and to protect and nurture microbial symbionts. Ludington [12] specifically describes the unique physical niches that enable these interactions, and delves into the ways that microbe-microbe interactions rely on region, order of arrival and host–microbe interactions.

Fujita *et al*. [13] extend this discussion on the interplay of host and microbe physiology. In their study, they develop a new technique to allow visualization of gut peristalsis inside *Drosophila,* without sacrificing animals. They then apply this technique to identify the microbe-derived metabolite acetylcholine as an inducer of host gut peristalsis. This study offers both a useful technical advance, and uses an elegant mix of metabolomics and host genetic tools to explore how microbe products impact host physiological state.

Interactions between hosts and bacterial metabolites are appreciated across organisms. Singh & Luallen [14] review factors shaping interactions between the roundworm *Caenorhabditis elegans* and its microbiome, including behaviour, immunity, diet and the gut niche. They underscore the use of this *in vivo* model in exploring the genetic basis and heritability of microbiome interactions with host health and disease, insights which may be broadly applicable to understanding host-microbiome interactions in animals including humans.

These host–microbiome interactions may be affected by changing climates for both hosts and the sources of their selected microbiomes. In this context, Li *et al*. [15] evaluated microbiomes of several wild *C. elegans* isolates across the globe and explored how warming temperatures and infection by the parasite *Leucobacter* change bacterial community structures. They found that parasite infection and warming independently and simultaneously destabilized host microbiomes, with parasitic infection causing the largest changes in dominant bacterial strains. Efforts like these are necessary to predict host population persistence in the wild under multiple stressors faced by all animals and microbes in an increasingly changing climate.

Thus far the issue has taken an experimental view of host–microbe and microbe–microbe relationships. Franz *et al*. [16] provide a unique perspective of how microbe-microbe interactions play out in the host given different infectious states. Their modelling study provides a basis for the evolutionary trajectories of the virulence of pathobionts, commensal microbes that can act in a virulent fashion upon disruption of host homeostasis. Franz *et al*. [16] explore the biologically realistic and woefully under-studied situation of co-infection, asking how the virulence of the initiating pathogen, the pathobiont and the overall system is shaped by different parameters of the infectious process.

This issue largely uses the language and framework established by the broader immunity, development, symbiosis and microbiome communities. Reflecting on this, the issue closes with an essay by Bosch *et al*. [17] on the language we use to frame studies of host–microbe interactions. Host–microbe interactions have long adopted the language of war, situated in a framework of host–pathogen conflict. Bosch *et al*. [17] provide a broader scope with which to view host–microbe systems, and propose updates to the lexicon we use that better acknowledge the diverse and content-dependent roles that host genes and microbial factors have in organismal health.

## 3. Conclusion

In the last decade, the precision offered by modern genetic tools has generated major advances in understanding how hosts select for, shape, and respond to the presence of the microbiome. We hope the perspectives in this issue will inspire new ways of thinking about how animals perform a microbial ‘sorting of the wheat from the chaff’ across stages of life and different physiological contexts. In the face of emerging crises such as antimicrobial resistance and global pandemics, an intimate understanding of how organisms detect and control microbes will be essential to respond to a rapidly changing microbial landscape.

It is well-appreciated that animals are not passive players in the microbial communities they associate with. Instead, this issue highlights the artistry of diverse organisms in sculpting and shaping their microbial associates. The product, the concept of ‘organism’ itself, is a composite of host factors and associated microbes. This issue can be viewed for its broad strokes, reflecting the powerful and universal underlying principles of host–microbe interactions. However, the reader is also encouraged to inspect more closely, appreciating the finer details and techniques used to refine these incredible sculptures. The organisms studied within represent the result of ages-old evolutionary processes that are yet ongoing. As curators of this exhibit in the museum of life most diverse, we hope the readers, the patrons, come away as inspired by the richness within as we are.

## Data Availability

This article has no additional data.

## Endnote

^1^
A framing provided by Kevin Kohl 2023, personal communication.
